# Grading of clear cell renal cell carcinoma using diffusion MRI with a multimodal apparent diffusion model

**DOI:** 10.3389/fonc.2025.1507263

**Published:** 2025-03-20

**Authors:** Shuang Wang, Tuo Ji, Dan Yu, Yimeng Dai, Butian Zhang, Lin Liu

**Affiliations:** ^1^ Department of Radiology, China-Japan Union Hospital of Jilin University, Changchun, Jilin, China; ^2^ Urology Department 1st Inpatient Area, China-Japan Union Hospital of Jilin University, Changchun, Jilin, China; ^3^ Department of MR Research Collaboration, United Imaging Research Institute of Intelligent Imaging, Beijing, China

**Keywords:** multimodal apparent diffusion model, clear cell renal cell carcinoma, World Health Organization/international society of urological pathology grading system, magnetic resonance imaging, diffusion-weighted imaging

## Abstract

**Objective:**

To assess the feasibility of utilizing parameters derived from a multimodal apparent diffusion (MAD) model to distinguish between low- and high-grade clear cell renal cell carcinoma (ccRCC).

**Method:**

Diffusion-weighted imaging (DWI) scans with 12 b-values (0 - 3000 s/mm²) were conducted on 54 patients diagnosed with ccRCC (30 low-grade and 24 high-grade). The MAD model parameters, including diffusion coefficients (D_r,_ D_h_, D_ui_, D_f_) representing restricted diffusion, hindered diffusion, unimpeded diffusion, and flow, respectively, were computed. Proportions corresponding to these diffusion types (f_r_, f_h_, f_ui_, f_f_) and the heterogeneous nature of hindered diffusion (α_h_) were also obtained. Parameters were compared between low- and high-grade groups. Receiver operating characteristic (ROC) curves were used to evaluate the diagnostic performance of these parameters, compared with the apparent diffusion coefficient (ADC) from a mono-exponential model.

**Result:**

Significant differences between low- and high-grade ccRCC were observed in D_h_ (low-grade: 1.360 ± 0.11 μm^2^/ms; high-grade group, 1.254 ± 0.13 μm^2^/ms; P = 0.0327), f_r_ (low-grade: 0.06 ± 0.005; high-grade: 0.08 ± 0.009; P = 0.0233), and α_h_ (low-grade: 0.872 ± 0.22; high-grade: 0.896 ± 0.39; P = 0.0294). Additionally, the ADC values (low-grade: 0.924 ± 0.08 μm^2^/ms; high-grade group, 0.854 ± 0.04 μm^2^/ms; P = 0.0323) showed statistical significance. The combination of D_h_, f_r_, and α_h_ provided the highest diagnostic accuracy of 0.667, with a sensitivity of 0.750, specificity of 0.734, and area under the curve of 0.796, outperforming individual parameters and ADC.

**Conclusion:**

The MAD diffusion model shows promise as a non-invasive imaging tool for distinguishing between low- and high-grade ccRCC, which may aid in preoperative planning and personalized treatment strategies.

## 1 Introduction

Clear cell renal cell carcinoma (ccRCC) represents the predominant malignant kidney tumor, accounting for approximately 90% of renal cancers globally (1). Despite surgical interventions such as radical or partial nephrectomy, around 30% of ccRCC patients experience metastasis or recurrence (2). Pathological features, including tumor stage, nuclear grade, necrosis, and lymphovascular invasion, are critical predictors of recurrence and patient survival (3). Among these, tumor grading is a vital prognostic factor, with the World Health Organization/International Society of Urological Pathology (WHO-ISUP) grading system categorizing ccRCC into four grades, ranging from low to high malignance (4). Patients with low-grade tumors (grade 1 and 2) typically have a longer tumor-free survival period compared to those with high-grade tumors (grade 3 and 4) (5). However, this grading system is traditionally reliant on invasive biopsy or post-surgical histopathological analysis, with preoperative biopsy often yielding unsatisfactory accuracy (6). This underscores the urgent need for a non-invasive technique capable of accurately grading ccRCC preoperatively.

Diffusion-weighted imaging (DWI) has gained widespread application in neuroimaging, body imaging, and oncological imaging. It exploits the disruption of water distribution between intracellular and extracellular compartments caused by pathological processes to generate detectable signals (7). Numerous studies have utilized DWI to evaluate the pathological characteristics of solid tumors, including grading, microvascular invasion, and subtype identification (8–10). Although the apparent diffusion coefficient (ADC) derived from a mono-exponential model provides insights into Gaussian water diffusion in tissues, it fails to capture the complexities of tissue microstructure, which are often indicative of tumor heterogeneity—a key characteristic of malignancy (11). This heterogeneity exists not only between individuals but also among different tumor cells within the same individual, leading to variations in tumor growth rate, invasion ability, drug sensitivity, and prognosis. Understanding this complexity is crucial for comprehending cancer intricacies and formulating effective treatment strategies (12). Currently, imaging techniques provide a non-invasive means to visualize tumors, revealing intuitive biological features such as size, shape, necrosis, bleeding, calcification, and vascular characteristics. Nonetheless, developing imaging models that capture microstructural complexity is essential for improving disease diagnosis, prognosis evaluation, and treatment planning.

Advanced diffusion models aim to address this limitation by leveraging variations in water molecule diffusion across different microstructures. Models like intravoxel incoherent motion (IVIM) and restriction spectrum imaging (RSI) segment diffusion signals into various components, providing a more nuanced representation of tissue complexity (13). Additionally, Restriction Spectrum Imaging (RSI) utilizes diffusion data collected from multiple gradient directions and b-values, along with an advanced linear mixture model, to segment the signal into hindered, restricted, and free water compartments. This approach provides estimates of specific tissue properties and explains the detailed decay of the diffusion signal (14).

The multimodal apparent diffusion (MAD) model, a novel approach, differentiates signals into flow, unimpeded, hindered, and restricted compartments, offering a comprehensive assessment of tissue heterogeneity (15). It takes into account that the voxel signal is the vector sum of synthesized complex signals of individual environments, which can be affected by the tissue’s current functional state. The comprehensive range from very low to high b-values (0 - 3000 s/mm²) was selected to capture both fast and slow diffusion components, thereby enhancing the reliability of the MAD model parameter estimations. By providing multiple parameters that can be correlated with tissue microstructure, the MAD model enhances the ability to interpret diffusion data in a biophysical context.

While MAD has shown promise in brain tumor studies (15), its application to body tumors, such as ccRCC, remains unexplored. This study aims to evaluate the feasibility of using MAD parameters for ccRCC grading and quantify tumor heterogeneity, comparing its effectiveness with the traditional mono-exponential model.

## 2 Methods

### 2.1 Patients

This study was approved by the Institutional Review Board (IRB) of the institution (No.2023103009). Between October 31, 2023(31/10/2023) and August 29, 2024(29/8/2024), patients presenting with suspected renal masses and subsequently undergoing MRI scans with multi b-value DWI were recruited. All participants provided informed consent by written prior to scanning. Post-surgical pathological assessment confirmed ccRCC diagnoses, with patients classified according to the WHO-ISUP grading system. The following exclusion criteria were applied: (i) Patients with contraindications for undergoing MRI examination, (ii) Individuals who had undergone invasive diagnostic procedures (e.g., biopsy or fine needle aspiration) or received chemotherapy, radiation therapy, or immunotherapy within the three days preceding the MRI examination, (iii) Patients who did not undergo surgical treatment within two weeks following the MRI examination, (iv) Cases where the quality of DWI images was unacceptable due to excessive artifacts or poor image quality rendering accurate evaluation impossible, (v) Patients whose pathological examination results revealed non-ccRCC diagnoses, including papillary renal cell carcinoma, pheochromocytoma, angiomyolipoma, eosinophilic tumors, and others.

### 2.2 Image acquisition

MRI examinations were conducted using a 3T scanner (uMR780, United Imaging Healthcare, Shanghai, China). Standard axial T1-weighted, axial fat-suppressed T2-weighted, and multi-b-value DWI sequences were acquired. DWI was performed using an echo planar imaging sequence with the following parameters: repetition time/echo time = 3000/58.4 ms, field of view = 380 mm, slice number = 24, slice thickness = 6 mm, intersection gap = 20%, matrix = 202×256, 12 b-values: 0(1), 20(1), 50(1), 100(1), 200(1), 500(2), 800(3), 1000(3), 1500(4), 2000(6), 2500(8), 3000 (9)s/mm^2^. The total scan time for DWI was 6 min 6s.

### 2.3 Image processing

All MAD data were post-processed using analysis software provided by the manufacturer. According to the MAD model, the voxel intensity in a diffusion-weighted image is given by the equation: (15)


$$\begin{array}{c} \frac{S(b)}{S(0)}=f_{r}\cdot \exp\;(-D_{r}\cdot b)+f_{h}\cdot \exp\;(-D_{h}\cdot b^{\alpha_{h}})+f_{ui}\cdot \exp\;(\\ -D_{ui}\cdot b)+f_{r}\cdot \exp\;(-D_{f}\cdot b), \end{array}$$

where D_r_, D_h_, D_ui_ and D_f_ are the diffusion coefficients representing restricted diffusion (D< 0.2 μm^2^/ms), hindered diffusion (D> 0.2 & < 3 μm^2^/ms), unimpeded diffusion (D≈3μm^2^/ms), and flow (D>> 3 μm^2^/ms), respectively. f_r_, f_h_, f_ui_ and f_f_ represent the proportions corresponding to these diffusion types, and α_h_ describes the heterogeneous nature of hindered diffusion. This approach enhances the characterization of tissue properties by minimizing the least squares difference between the model and the data, and by incorporating linear regression for increased efficiency and noise resilience. For the mono-exponential ADC model, linear fitting across all b-values (0 to 3000 s/mm²) is used. While using a wide range of b-values may introduce bi-exponential signal behavior, the aim is to standardize the ADC calculation for all subjects to facilitate direct comparisons.

The region of interest (ROI) was selected and drawn by two radiologists with 8 and 13 years of experience in abdominal MRI, who were blinded to the study design and diagnosis. The mean value from two readers’ measurements was adapted into further analysis. The target area was delineated on the maximum cross-section of the tumor, ensuring the inclusion of as much of the lesion area as possible while excluding internal areas of necrosis, calcification, and bleeding. The tumor ROIs were selected on the b=800 s/mm^2^ images due to its best distinction between lesions and the surrounding tissue and then delineated ROI contours were applied to the maps of MAD diffusion parameters and calculated on a voxel-by-voxel basis. Mean values of these parameters were then computed from the ROIs.

### 2.4 Statistical analysis

All data were analyzed using GraphPad Prism (Version 10.2.0). The Kolmogorov-Smirnov Test was applied to assess the normality of the data. Statistical data were expressed as mean ± standard deviation or median quartile. The mean value of each diffusion parameter over the ROI was computed, followed by comparisons between the low- and high-grade groups using independent-sample t-test. For data that did not conform to the normal distribution level or had uneven variances, the Mann-Whitney test was used. Effect sizes were calculated using Cohen’s d to assess the practical significance of differences between groups. Receiver operating characteristic (ROC) curves were established for each parameter with significance, using the area under the ROC curve (AUC) to measure the discriminative effect of various parameters of MAD model and ADC values between two groups. A P-value < 0.05 was considered statistically significant.

## 3 Results

### 3.1 Patients

**Figure 1:**
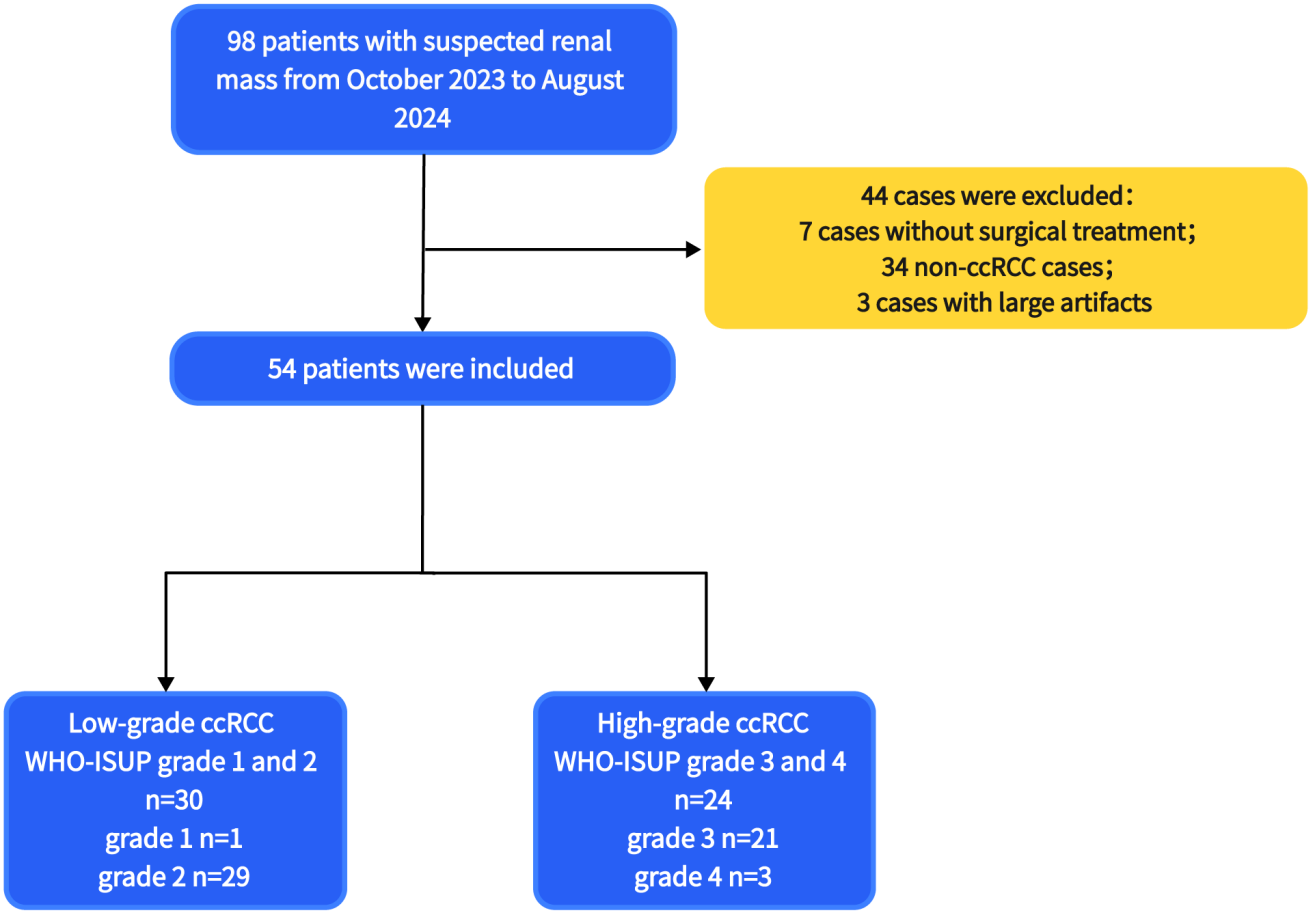
The flowchart of patients selection. ccRCC, clear cell renal cell carcinoma; WHO-ISUP, World Health Organization/International Society of Urological Pathology.

**Figure 2:**
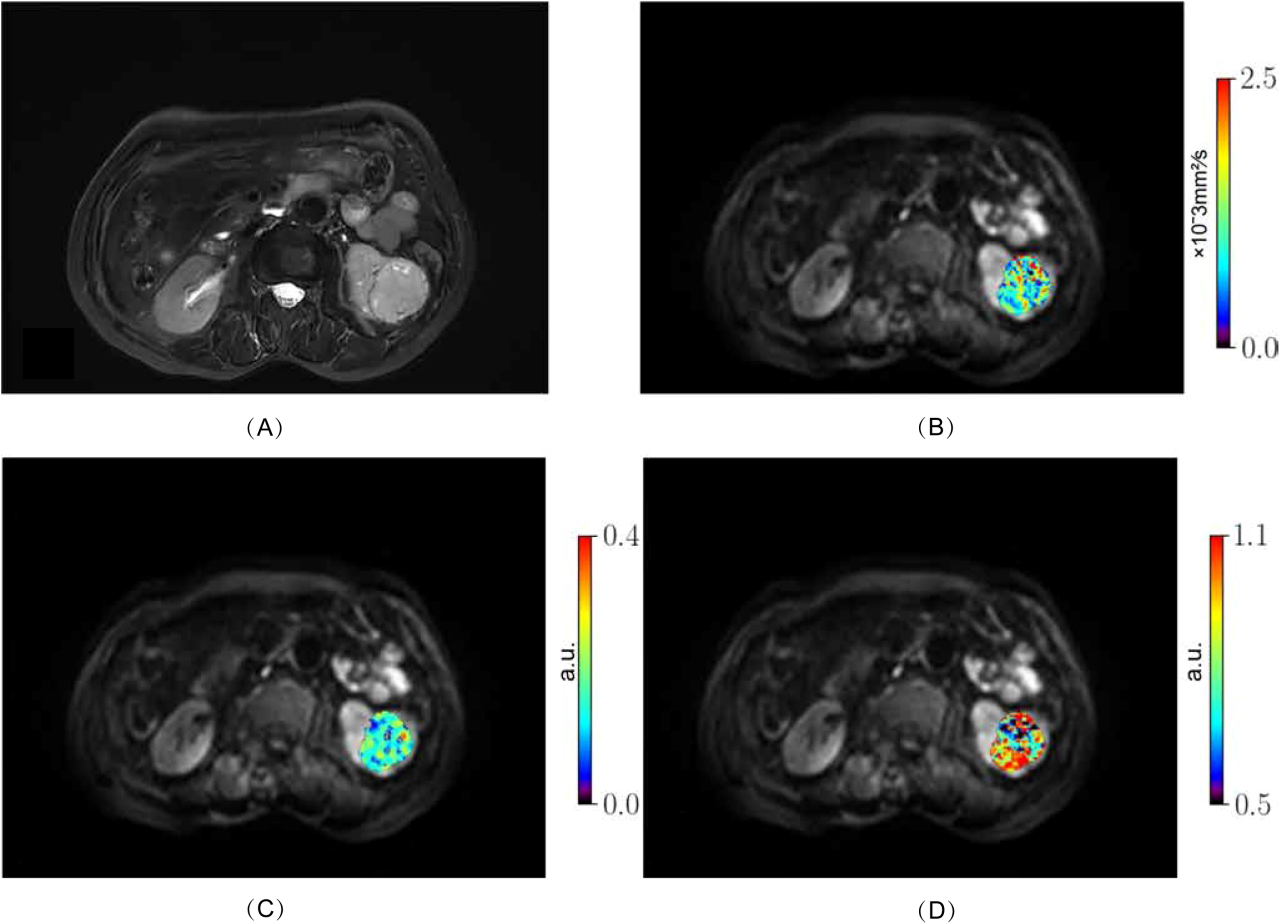
Low-grade ccRCC (WHO-ISUP grade 2) in the left kidney of a 76-year-old woman. **(A)**, axial fat-suppressed T2-weighted image; **(B–D)** MR images with quantitative MAD maps: **(B)**, D_h_ values; **(C)**, f_r_ values and **(D)**, α_h_ values. ccRCC, clear cell renal cell carcinoma; MAD, multimodal apparent diffusion model.

**Figure 3:**
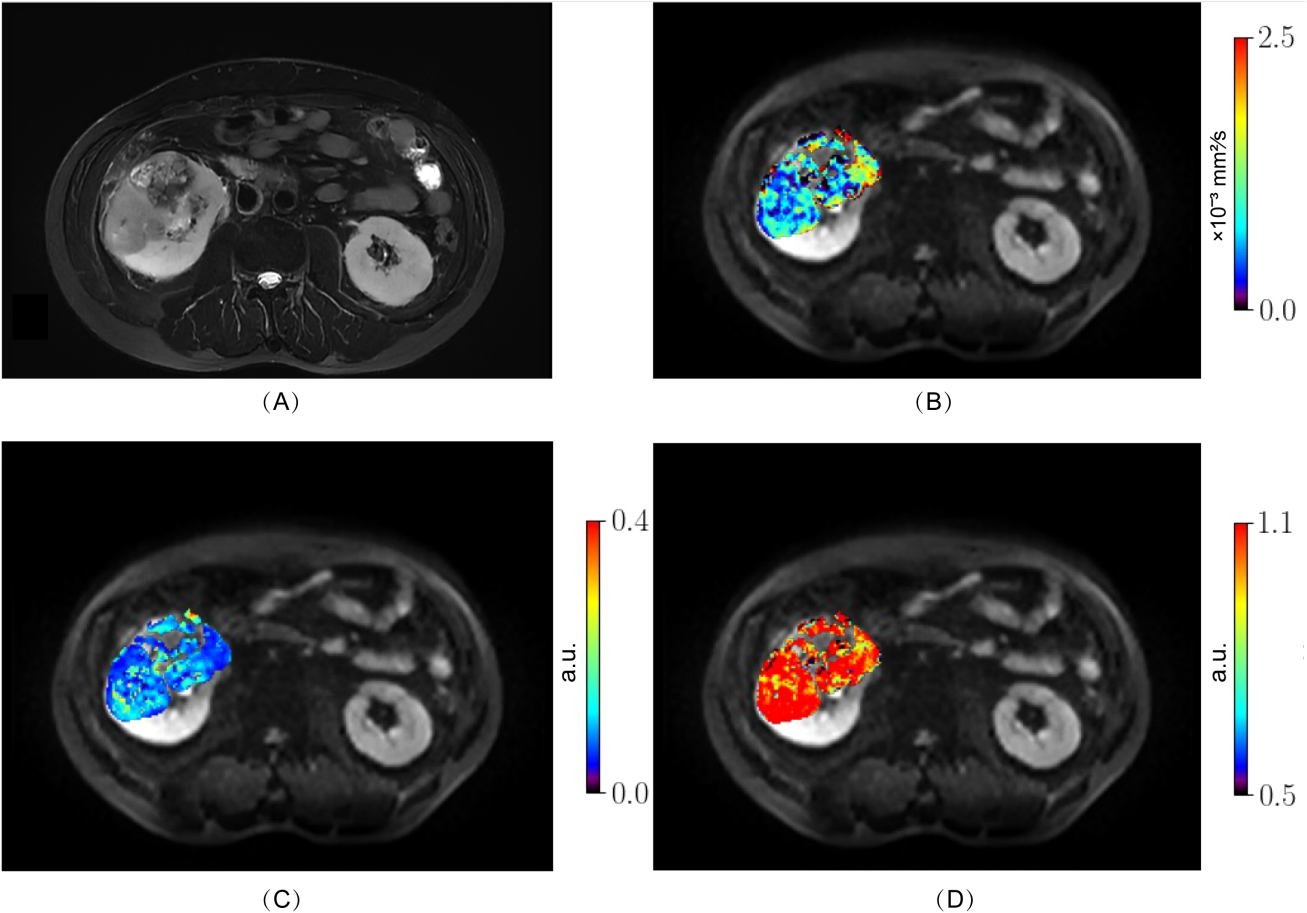
High-grade ccRCC (WHO-ISUP grade 4) in the right kidney of a 50-year-old man. **(A)**, axial fat-suppressed T2-weighted image; **(B–D)** MR images with quantitative MAD maps. **(B)**, D_h_ values; **(C)**, f_r_ values and **(D)**, α_h_ values. The MAD maps **(B–D)** show substantial differences between low- (**Figure 2**) and high-grade tumor (**Figure 3**). ccRCC, clear cell renal cell carcinoma; MAD, multimodal apparent diffusion model.

From October 2023 to August 2024, 98 patients with suspected renal masses were enrolled. All patients underwent MRI examination, including multiple b-value DWI within 3 days, without undergoing invasive examination (such as biopsy or fine-needle aspiration), chemotherapy, radiotherapy, or immunotherapy. Informed consent was obtained from all participants before examination. Out of these, 91 patients underwent surgical treatment within two weeks, and 7 patients did not undergo surgical treatment. Pathological examination confirmed 57 cases of ccRCC, while 14 cases of papillary renal cell carcinoma, 5 cases of chromophobe cell carcinoma, 8 cases of angiomyolipoma, and 7 cases of eosinophilic cell tumor were excluded. Additionally, 3 cases of ccRCC were excluded due to large artifacts or poor image quality. Finally, a total of 54 cases (38 men, 16 women; median age 68 years; range 38 - 76 years) of ccRCC patients were included. According to the WHO-ISUP grading system, ccRCC was divided into four grades. Low-grade (WHO-ISUP grade 1 and 2, n = 30; grade 1, n = 1; grade 2, n = 29; 18 men, 12 women; median age 61 years; range 38 - 76 years) and high-grade (WHO-ISUP grade 3 and 4, n = 24; grade 3, n = 21; grade 4, n = 3; 20 men, 4 women; median age 62 years; range 42 - 74 years) were defined for subsequent research (**Figure 1**). The Clinical Characteristic is shown in **Table 1**. **Figures 2**, **3** show a set of images obtained from 2 representative patients with low- and high-grade tumor (WHO-ISUP 2 and WHO-ISUP 4 grade ccRCC) respectively.

**Table 1:** Clinical characteristics of patients.

Characteristics	All patients (n=54)	Low-grade (n=30)	High-grade (n=24)
Age (years)			
Median	68	61	62
Range	38-76	38-76	42-74
Sex			
Male	38 (70.4)	18 (60.0)	20 (83.3)
Female	16 (29.6)	12 (40.0)	4 (16.7)
WHO/ISUP grade			
Grade 1	1 (1.9)	1 (3.3)	0 (0)
Grade 2	29 (53.7)	29 (96.7)	0 (0)
Grade 3	21 (38.9)	0 (0)	21 (87.5)
Grade 4	3 (5.6)	0 (0)	3 (12.5)
T stage	(n=54)	(n=30)	(n=24)
T1	34 (63.0)	26 (86.7)	8 (33.3)
T2	3 (5.6)	0 (0)	3 (12.5)
T3	14 (25.9)	3 (10.0)	11 (45.8)
T4	3 (5.6)	1 (3.3)	2 (8.3)

Percentages are based on group totals (All: n=54, Low-grade: n=30, High-grade: n=24). WHO/ISUP, World Health Organization/International Society of Urological Pathology. T stage: Tumor stage classification (T1-T4). Data are presented as n (%).

### 3.2 Diffusion metrics

**Figure 4:**
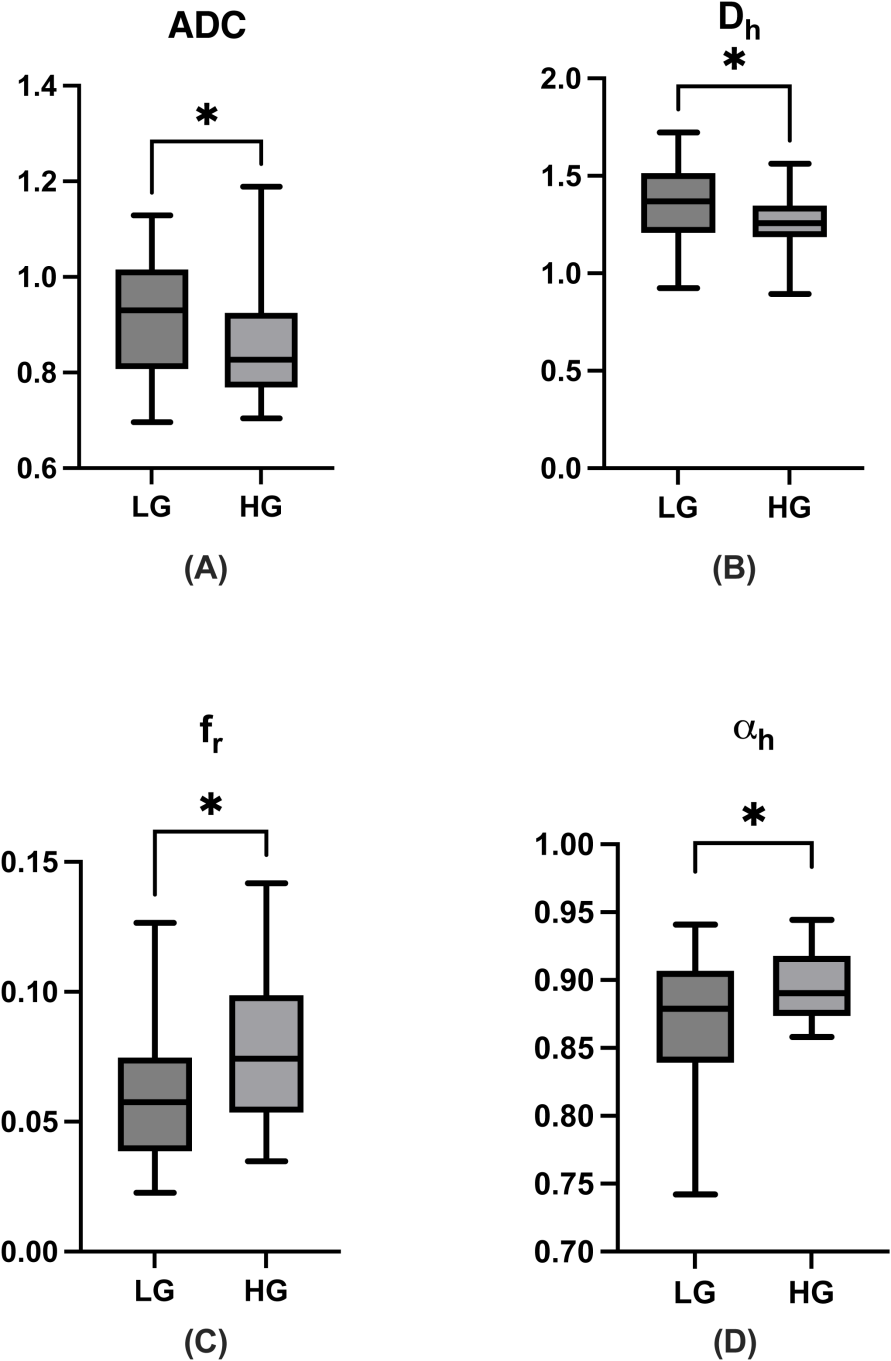
Boxplots of ADC, D_h_, f_r_, and α_h_ values between low- and high-grade ccRCC **(A–D)**. ADC, apparent diffusion coefficient; ccRCC, clear cell renal cell carcinoma; LG, low- grade clear cell renal cell carcinoma; HG, high-grade clear cell renal cell carcinoma. The symbol “*” represents statistically significant differences.

Among all parameters, the values of D_h_, f_r,_ and α_h_ derived from MAD model and the values of mono-exponential ADC demonstrated statistical significance in distinguishing between low- and high- grade ccRCC. The D_h_ values of high-grade group were significantly lower than those of low-grade group (low-grade group, 1.360 ± 0.11 μm^2^/ms; high-grade group, 1.254 ± 0.13 μm^2^/ms; P = 0.0327). The f_r,_ values were significantly higher in the high-grade group than those in the low-grade group (low-grade group, 0.06 ± 0.005; high-grade group, 0.08± 0.009; P = 0.0233). The values of α_h_ were also with statistical significance between low- and high-group (low-grade group, 0.872 ± 0.22; high-group, 0.896 ± 0.39; P = 0.0294). Additionally, the ADC values of the high-grade group were significantly lower compared to those of the low-grade group (low-grade group, 0.924± 0.08μm^2^/ms; high-grade group, 0.854± 0.04 μm^2^/ms; P = 0.0323). Other MAD model parameters did not show significant differences between the two groups (f_h_, P = 0.3569; f_ui_, P=0.7436; f_f,_ P = 0.2942; D_r_, P = 0.3750; D_f_, P = 0.9809). The results were shown in **Table 2** and **Figure 4**. Effect sizes (Cohen’s d) were calculated for D_h_ (d = 0.888), f_r_ (d = 2.835), and ADC (d = 1.070), indicating moderate to large practical significance.

**Table 2:** Values of MAD model diffusion parameters and values of mono-exponential ADC for low- and high-grade ccRCC.

Parameter	Low-grade group	High-grade group	P value
D_r_(μm^2^/ms)	0.039(0.014,0.072)	0.045(0.033,0.069)	0.3750
D_h_(μm^2^/ms)	1.360 ± 0.112	1.254 ± 0.134	0.0327*
D_f_(μm^2^/ms)	15.012 ± 1.8	14.335 ± 1.2	0.689
f_r_(unitless)	0.060 ± 0.005	0.080± 0.009	0.0233*
f_h_(unitless)	0.413 ± 0.029	0.442 ± 0.031	0.357
f_ui_(unitless)	0.275(0.224,0.373)	0.274(0.230,0.320)	0.743
f_f_(unitless)	0.219 ± 0.023	0.196 ± 0.022	0.294
α_h_(unitless)	0.872 ± 0.224	0.896 ± 0.393	0.0294*
ADC(μm^2^/ms)	0.924± 0.081	0.854± 0.045	0.0323*

Statistical data were expressed as mean ± standard deviation or median and interquartile range. The last column displays the p values for the difference in the parameters between the low- and high-grade groups by using independent-sample t-test or Mann-Whitney test. A p-value less than 0.05 is statistically significant with ‘*’ in the upper right corner.

ADC, apparent diffusion coefficient; ccRCC, clear cell renal cell carcinoma; MAD, multimodal apparent diffusion.

### 3.3 Diagnostic performance of diffusion metrics

**Figure 5:**
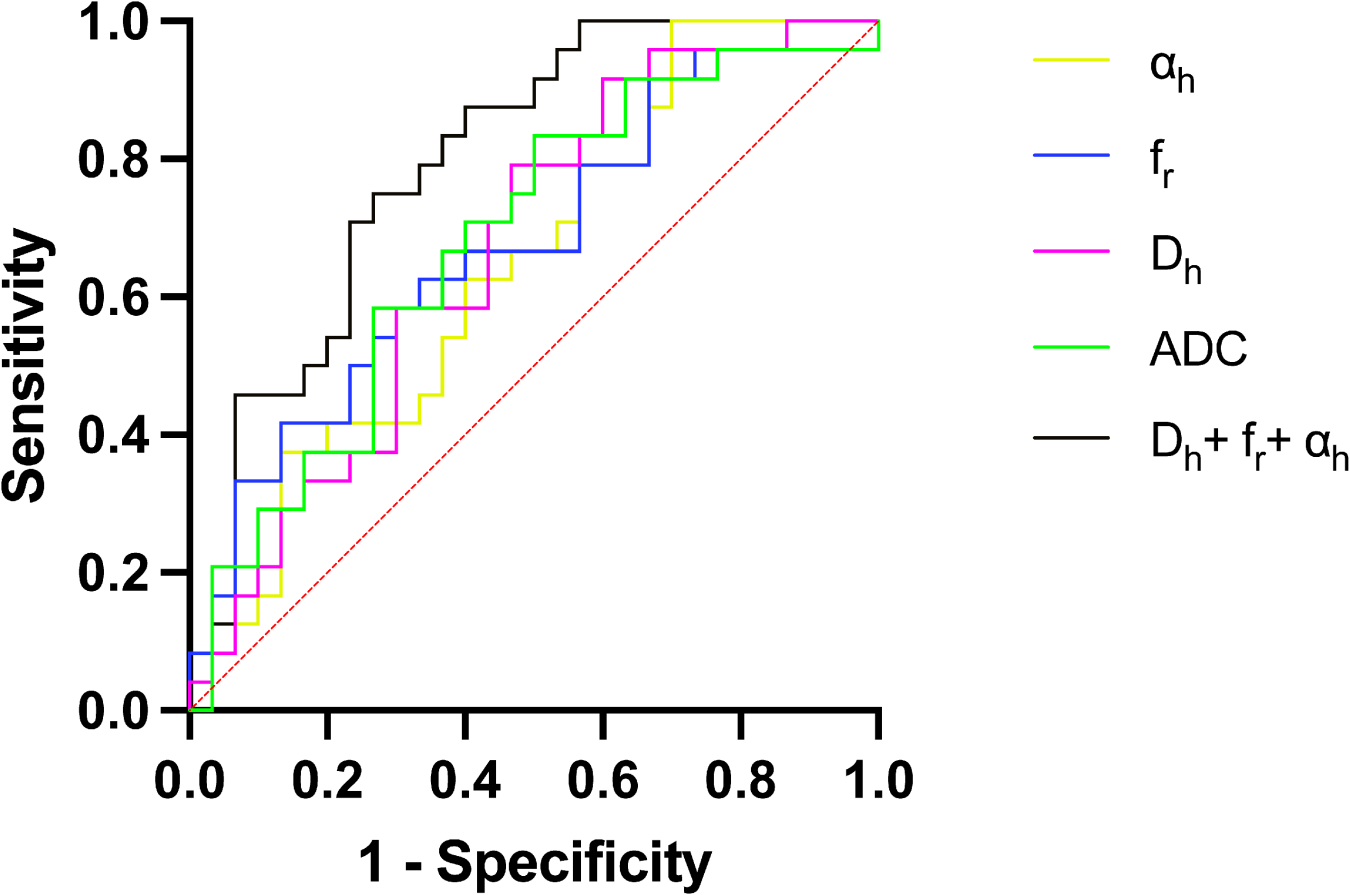
ROC curves for D_h_, f_r_, α_h_, the combination of D_h_, f_r_ and α_h_, and ADC for differentiating low- and high-grade ccRCC. ADC, apparent diffusion coefficient; AUC, area under the curve; ccRCC, clear cell renal cell carcinoma; ROC curve, Receiver operating characteristic curve.

**Figure 5** shows the ROC curves and **Table 3** show the results from the ROC analysis for predicting the grading of ccRCC using statistically significant parameters of MAD model, namely D_h_, f_r_, α_h_ and their combination, compared with the ADC value. The multiple logistic regression model established by combination of D_h_, f_r_ and α_h_ yield the best diagnostic performance in accuracy of 0.667 and AUC of 0.796. This was followed by ADC (accuracy, 0.648; AUC, 0.681), f_r_ (accuracy, 0.630; AUC, 0.681), D_h_, (accuracy, 0.593; AUC, 0.668) and α_h_ (accuracy, 0.556; AUC, 0.646). More details about the diagnostic performance of the quantitative indexes are listed in **Table 3**.

**Table 3:** The ROC analysis results of using MAD model parameters and ADC to grade clear cell renal cell carcinoma.

Statistics	ADC	D_h_	f_r_	α_h_	D_h_+ f_r_+α_h_
Sensitivity	0.833	0.917	0.625	0.958	0.750
Specificity	0.500	0.400	0.667	0.300	0.734
Accuracy	0.648	0.593	0.630	0.556	0.667
AUC	0.681	0.668	0.681	0.646	0.796
95%CI	0.537-0.825	0.524-0.812	0.537-0.824	0.499-0.792	0.677-0.914

ADC, apparent diffusion coefficient; AUC, area under the ROC curve; CI, confidence interval, MAD, multimodal apparent diffusion.

## 4 Discussion

This study explored the feasibility of using the multimodal apparent diffusion (MAD) model to distinguish between low- and high-grade ccRCC. The results indicated that D_h_, f_r_, and α_h_ are statistically significant in differentiating between low- and high-grade ccRCC. Additionally, multivariate logistic regression analysis showed that combining these parameters can improve the accuracy of grade prediction. These findings highlight the potential of MAD models for preoperative grading of renal cancer.

The mono-exponential model, which assumes free water motion in tissues, cannot fully capture the complexities of tumor tissues, particularly their heterogeneity (16). Tumor tissues exhibit significant heterogeneity within the lesion. The MAD model can describe four diffusion modes of water molecules in tissues: restricted diffusion, hindered diffusion, unimpeded diffusion, and flow. By integrating intravoxel incoherent motion (IVIM) and the Stretched-Exponential Model (SEM), the MAD model considers both high and low b-values, facilitating the evaluation of cell structure, microstructure, and tissue heterogeneity. This capability is particularly useful in tumor tissues and may have implications for tumor grading and prognosis. By providing multiple parameters that can be correlated with tissue microstructure, the MAD model enhances the ability to interpret diffusion data in a biophysical context, which is particularly useful in tumor tissues and may have implications for tumor grading and prognosis.

In this study, ADC in the mono-exponential model and the D_h_ parameter in the MAD model showed statistical significance in distinguishing between low- and high-grade ccRCC, consistent with the findings of previous studies by Rosenkrantz et al. (17) and Shen et al. (18). These results suggest that high-grade tumors impose greater restrictions on water molecules within their microstructure compared to low-grade tumors (17). In MAD model, the diffusion coefficient in low- and high-grade tumors is predominantly influenced by hindered diffusion, with an apparent diffusion coefficient ranging from approximately 0.1 to 3 μm^2^/ms. This suggests that high-grade tumors possess smaller intercellular spaces, more tightly interconnected cells, heightened expression of various extracellular proteins and matrices, and more rapid cell division and enlargement (17).

Although the proportion of f_r_ is relatively small, the f_r_ was significantly higher in high-grade ccRCC compared to low-grade tumors. The f_r_ refers to the proportion of water under Brownian motion that had reached an impulse within Δ, resulting in an apparent diffusivity was much smaller than μm^2^/ms, mainly representing the proportion of restricted diffusion of water molecules within cells (15). The WHO-ISUP grading system primarily focuses on the prominence of nuclei, with the first three grades determined by nucleolar size. As the grade of ccRCC increases, the nuclear volume also enlarges. Grade 4 tumors are characterized by extreme nuclear pleomorphism, including atypical tumor giant cells and/or sarcomatoid/rhabdoid differentiation (19). These nuclear enlargement and morphological changes decrease intracellular space, leading to restricted diffusion of water molecules. Additionally, studies have confirmed that the accumulation of cellular proteins within cells is positively correlated with the grading of ccRCC (19). As the grade of ccRCC escalates, the intracellular movement of water molecules becomes increasingly constrained, ultimately resulting in restricted diffusion, which closely aligns with the hallmarks of pathological grading. Consequently, this metric holds significant potential as a parameter for both quantifying and visually representing the grading of ccRCC.

The α_h_ plays a pivotal role in distinguishing between low- and high-grade ccRCC. Specifically, the α value quantifies the extent to which signal decay within a voxel deviate from a mono-exponential decay. In this study, higher α_h_ values were observed in high-grade ccRCC compared to low-grade ccRCC. This finding is counterintuitive since high-grade tumors are generally more heterogeneous. The α_h_ parameter in the context of the MAD model corrects for one compartment of diffusion heterogeneity, primarily hindered diffusion. While the D_h_ parameter models hindered diffusion, α_h_ captures the remaining inhomogeneity after accounting for this component. Higher α_h_ values in high-grade tumors may indicate that the microenvironment, although biologically complex, has a more uniform structural organization compared to low-grade tumors. High-grade tumors often exhibit increased cellular density and more organized extracellular matrix structures. This can lead to a more uniform appearance in diffusion imaging, even though the tumor’s biological behaviour is more aggressive. Studies on other cancer types using similar diffusion models and texture analysis have shown that high-grade tumors can display lower entropy and higher uniformity in imaging parameters, which parallels the higher α_h_ values observed in this study (20).

The MAD model has been applied to brain tumors, where D_h_ (hindered diffusion coefficient) and f_r_ (restricted diffusion fraction) reflect microstructural properties. In gliomas, D_h_ is influenced by cell density and edema, while f_r_ correlates with necrosis or hypercellularity (15). In ccRCC, D_h_ likely reflects tightly packed epithelial cells and extracellular matrix, while elevated f_r_ in high-grade ccRCC aligns with nuclear enlargement and intracellular protein accumulation. In brain tumors, α_h_ reflects heterogeneity from infiltrative growth or vasogenic edema, whereas in ccRCC, α_h_ is better interpreted as the shape parameter of restricted diffusion, indicating the narrowness or uniformity of the diffusion distribution. Increased cell density and structural complexity in high-grade tumors may result in a more uniform diffusion pattern, leading to higher α_h_ values. These differences highlight the need for tissue-specific calibration when applying the MAD model to different organ systems.

Although there was some overlap in diffusion metrics between low- and high-grade tumors, the effect sizes indicate that the differences have moderate to large practical significance, supporting the clinical relevance of these findings.

The limitations of this study include the relatively small number of high-grade ccRCC patients, primarily due to the early detection of many tumors through screening, which may have biased the results and limited statistical power. Future research should aim to include a larger sample size to validate these findings. Additionally, the ROI was chosen based on the largest solid region of the tumor without considering all tumor levels, which may have introduced bias. A more comprehensive approach to ROI selection could provide a more accurate representation of the tumor’s heterogeneity. Another limitation is the uneven distribution of patients across different grades, with the majority falling into grade 2 or 3. This uneven distribution may have reduced the statistical differences between groups, impacting the diagnostic utility of diffusion indicators. Furthermore, histopathological data such as cell count or Ki67 were not utilized to establish their correlation with diffusion parameters. Future research should incorporate these data to better understand the relationship between histopathological characteristics and diffusion metrics. Furthermore, while we aimed to interpret each MAD parameter with biophysical explanations, we did not directly compare our findings with pathological results. Future studies should incorporate histopathological correlations to enhance the understanding of how MAD parameters relate to specific tissue characteristics. Lastly, the exploratory nature of this study involved multiple comparisons without correction, increasing the risk of Type I errors. However, we focused on parameters with the most clinical relevance and calculated effect sizes to assess practical significance. Although the initial analysis aimed to identify potential significant parameters, future research should include corrections for multiple comparisons, such as the Bonferroni or False Discovery Rate adjustments, to ensure the robustness of the findings.

## 5 Conclusion

In conclusion, this study demonstrates that the MAD diffusion model significantly improves the differentiation between low- and high-grade ccRCC compared to the conventional ADC model. By providing multiple diffusion parameters that correlate with tumor microstructure, the MAD model offers a more comprehensive and nuanced assessment of tumor grade. The association between MAD parameters and tumor grading suggests that this model has the potential to serve as a reliable non-invasive imaging biomarker for ccRCC grading. Implementing the MAD model in clinical practice could enhance preoperative assessment, guide personalized treatment strategies, and ultimately improve patient outcomes. Further large-scale studies with histopathological correlations are warranted to validate our findings and fully establish the clinical utility of the MAD diffusion model in renal tumor grading.

## Data Availability

The original contributions presented in the study are included in the article/supplementary material. Further inquiries can be directed to the corresponding authors.
